# No wisdom in the crowd: genome annotation in the era of big data – current status and future prospects

**DOI:** 10.1111/1751-7915.13284

**Published:** 2018-05-28

**Authors:** Antoine Danchin, Christos Ouzounis, Taku Tokuyasu, Jean‐Daniel Zucker

**Affiliations:** ^1^ Integromics Institute of Cardiometabolism and Nutrition Hôpital de la Pitié‐Salpêtrière 47 Boulevard de l'Hôpital 75013 Paris France; ^2^ School of Biomedical Sciences Li KaShing Faculty of Medicine Hong Kong University 21 Sassoon Road Pokfulam Hong Kong; ^3^ Biological Computation and Process Laboratory Centre for Research and Technology Hellas Chemical Process and Energy Resources Institute Thessalonica 57001 Greece; ^4^ Shenzhen Institutes of Advanced Technology Institute of Synthetic Biology Shenzhen University Town 1068 Xueyuan Avenue Shenzhen China

## Abstract

Science and engineering rely on the accumulation and dissemination of knowledge to make discoveries and create new designs. Discovery‐driven genome research rests on knowledge passed on via gene annotations. In response to the deluge of sequencing big data, standard annotation practice employs automated procedures that rely on majority rules. We argue this hinders progress through the generation and propagation of errors, leading investigators into blind alleys. More subtly, this inductive process discourages the discovery of novelty, which remains essential in biological research and reflects the nature of biology itself. Annotation systems, rather than being repositories of facts, should be tools that support multiple modes of inference. By combining deduction, induction and abduction, investigators can generate hypotheses when accurate knowledge is extracted from model databases. A key stance is to depart from ‘the sequence tells the structure tells the function’ fallacy, placing function first. We illustrate our approach with examples of critical or unexpected pathways, using MicroScope to demonstrate how tools can be implemented following the principles we advocate. We end with a challenge to the reader.

## Introduction

‘Data make no sense!’ fumed Noam Chomsky, back in 1974 at the MIT's Endicott House, during a meeting of the Centre Royaumont pour une Science de l'Homme on Brain and Cognition, putting an end to a talk that had featured linguistic data. He was right. Data may make sense as a diagnostic tool – this is the major way data are used unscrupulously on the World Wide Web in particular for commercial purposes. In those situations, it does tell something, but not about the things you are looking for, rather about yourself. And if you know both how to use behaviour as a monitor to predict some future outcome you know exactly what to do. Yet, in our time of big data and within the academic realm, scientists, not merchants, keep producing a wealth of data under collective names ending in ‘‐ome’ or ‘‐omics’ supposed to be the ultimate way to decipher what life is – an implicit assumption, perhaps and, ironically, also driven by commercial interests. Armed with arcane techniques with some mathematical flavour, researchers attempt to have conceptual knowledge emerge magically out of these huge data‐collecting efforts. Three major approaches are used to this goal: knowledge‐driven, data‐driven or context‐driven (Bolton, 2015), strikingly following specific trends of human language principles (represented by Greco‐Latin, Anglo‐American or Chinese linguistic families respectively). Knowledge‐driven approaches entail deduction, *i.e*. based on a hypothesis derived from previously acquired knowledge, – for example if someone is exposed to a factor which we regard as a health risk, then they might fall ill. Data‐driven approaches entail induction, *i.e*. starting from a fact, we can infer a general trend – for example if some factor is a health risk, and we meet patients exposed who suffer from the relevant health issue, we can infer that these patients were exposed to that factor. Finally, context‐driven approaches entail abduction [(Ferneda *et al*., 1995) and references therein]. A crude view is to see this approach as a trial‐and‐error method familiar only to those who have delved into artificial intelligence – for example facing a forest at night you shoot in the dark, if you hear a cry, then you have a handle to start exploring; if not, you try again.

However, conceptual progress is not linked solely to the data – in fact, data might indeed form a burden. Conceptual processing results in metadata in a manner that is not always explicit. For instance, the process of genome annotation links local sequences to metadata such as biochemical information. As this fundamentally connects genotypes to phenotypes, this is highly relevant to biological enquiry. This calls for a specific prerequisite: we must have initiated data collection with a scientifically meaningful purpose in mind (that is, not solely aiming at facilitating diagnosis), which is meant to answer, as widely and accurately as possible, specific and valid questions posed at the onset of the experiment collecting the data – and definitely not a posteriori. This constraint strongly implies that highly relevant metadata should always be collected prior to data collection.

All the same, we are flooded by genome data merely because sequencing has become so accessible and cost‐effective (Schmidt and Hildebrandt, 2017), with significant, large‐scale efforts regularly making the headlines of popular dailies. In early days, when sequencing was extremely tedious, sequences were obtained with a specific view in mind, linking them in a straightforward way to relevant and rich meta‐information. With rapid progress and radical improvements of sequencing techniques, it became important to start collecting further informative annotations, some of which might be less obvious. The creation of databanks has always played a central role as reference repositories of all biological information that could be linked to DNA and protein sequences. This process of submission and re‐distribution was first performed manually until, not without some controversy, journals decided to start accepting articles containing novel sequence data. Their condition was that all molecular information would become publicly available so that data sharing would automatically feed into the collection process (Roberts and Koetzle, 1989; Blaxter *et al*., 2016). After a long series of discussions and efforts, this resulted in the establishment of the International Nucleotide Sequence Database Collaboration [INSDC (Karsch‐Mizrachi *et al*., 2018)], exchanging and updating sequences on a daily basis between three entry points, DDBJ in Japan (Kodama *et al*., 2018), ENA in Europe (Silvester *et al*., 2018) and GenBank in the United States (Benson *et al*., 2018).

As sequencing became cheaper and easier, it became manifest that manual annotation could not keep up with output and that one had to resort to automatic annotation [for early attempts, see (Staden, 1977; Gingeras and Roberts, 1980; Bossinger, 1988; Scharf *et al*., 1994)]. Unexpectedly, during the early days of sequencing, it became evident that sequence libraries already contained similar entries, usually collected via a common functional approach. Such observations led to the core idea that homologous sequences should code for similar structures and related functions. This working hypothesis has not been comprehensively tested, although it appears to generally have been correct with notable, and sometimes detrimental, exceptions.

As a consequence, it was essential to measure similarity in a fast and efficient way. This resulted in the unprecedented success of the BLAST program (Altschul *et al*., 1990), which, used under a variety of flavours, is still the most efficient and widely available way to compare sequences. Furthermore, similarities were grouped together using BLAST bidirectionally, creating the Clusters of Orthologs that are still in use (Galperin and Koonin, 1999), the TRIBEs resource (Enright *et al*., 2003) and others that followed. A family of tools, critical both for the identification of important sites in proteins and their evolution, allowed the multialignment of protein sequences (Lipman *et al*., 1989). These are now used in a large variety of software that rest on specific hypotheses monitoring similarities and evolution [see (Pearson *et al*., 2017; Zambrano‐Vega *et al*., 2017; Sievers and Higgins, 2018) for recent developments in multiple protein sequence alignment]. Further tools, in an unlimited number of flavours, are now used to investigate sequences with the aim of predicting their function, implicitly validating the inference: ‘sequence tells structure tells function’.

A key requirement remains that the functional output of all relevant methods should be connected to sequences. This implies that data are not the raw sequences but tidied up sequences already associated with method‐driven metadata and multiple classification schemes (Ouzounis *et al*., 2003). This requirement goes hand in hand with the essential compression step that has become key to streamline the huge amount of sequence data that is flooding computer memories (Cochrane *et al*., 2013). To sum up, data must be split and grouped into functionally relevant data families. This requires data structuration –that is further organization of data into appropriate structures (Wang *et al*., 2002; zu Siederdissen *et al*., 2015; Kruse *et al*., 2016 ).

## Data structures

Genome sequence data are strings of the four DNA nucleotide bases. Experimentally obtained, they are associated with a first range of metadata such as sequence quality, fragment length or methodology‐oriented paired‐ends sequencing (Bianchi *et al*., 2016). Subsequently, when sequences begin to be organized with the aim of specific biological understanding, we have long reads, contigs and also repeats, G+C content, tetranucleotide frequencies and a plethora of sequence descriptors [see (Weinel *et al*., 2002) as an example]. Furthermore, genome sequences are implicitly associated with available knowledge about biology, based on the notion that they must code for genes with their regulatory regions and especially protein coding sequences (CDSs). More recently, a large family of RNAs [regulatory ‘noncoding’ RNAs (Hor *et al*., 2018), riboswitches (Serganov and Nudler, 2013), ribozymes (Wilson and Lilley, 2015) and other miscellaneous RNAs (Nelson and Breaker, 2017)] completed the picture. As relevant metadata, this involved the tagging of gene sequences for control regions (Nikolaichik and Damienikan, 2016) as well as other elements of genome organization, and of course the source of the sequence, often a scientific article. Taken together, all these pieces of metadata must form an explicit data structure that will be recognized by a variety of database schemas.

**Figure 1:**
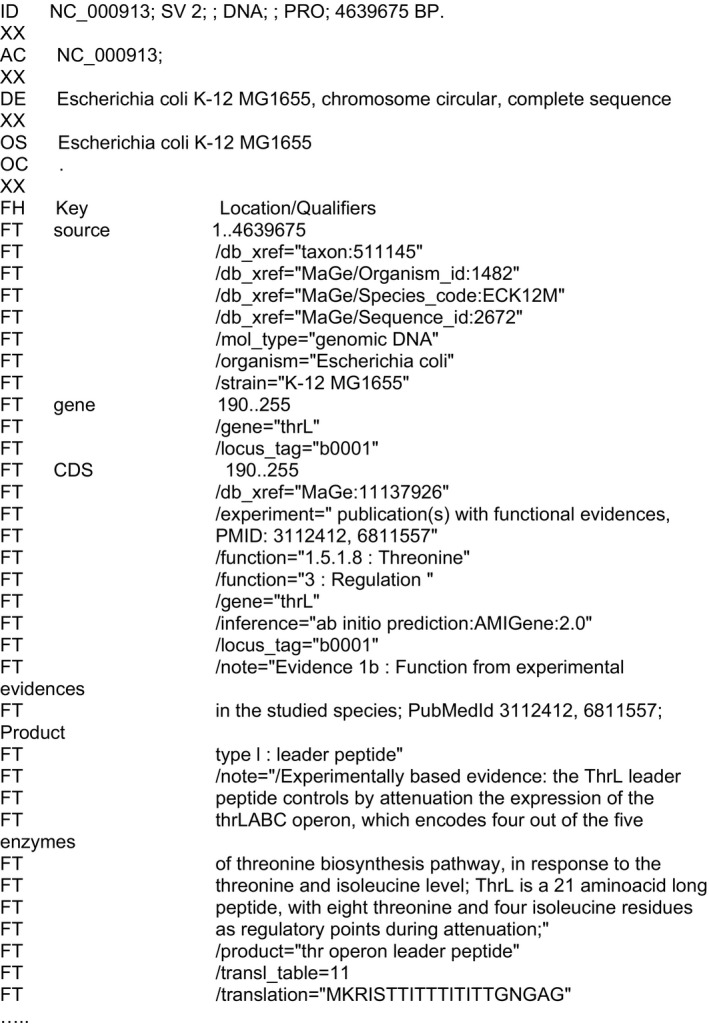
The beginning of the *Escherichia coli* model genome ENA record. Similar records are deposited at DDBJ and at the NCBI. The file goes through all annotated genomic objects (here a gene) with specific fields recording information associated with the sequence data. Throughout several decades of work, the INSDC partners progressively defined new fields in the record meant to inform the user about the information available at the time of the record depository.

The core data object associated with multiple metadata tags is usually a DNA sequence, the ‘genomic object’ (Fig. 1). However, in some databases the choice is different: the *Genes* section of FlyBase includes information on *Drosophila* genes that has been curated from the literature and sequence databases. In this way, the scientific article is the key field connecting other fields together in the database (Gramates *et al*., 2017). It should be obvious, then, that input and extraction of information from such diverse data collections will lead to widely different pathways to discovery. An inconspicuous but immense (ongoing) effort in this domain is undertaken by all participants of the INSDC (Karsch‐Mizrachi *et al*., 2018), who endeavour to set up data structures in order to collect and make freely available nucleotide data sequences described in scientific articles, patents or deposited directly at one of its three entry points (DDBJ, ENA, GenBank). An important point here is that how data is structured has a far‐reaching impact on data annotation quality. Data structures affect the ease with which a community of investigators can submit annotations. They can facilitate, or alternatively prevent, accurate annotation. Furthermore, discoveries made in experimental laboratories depend heavily on the quality of the data annotation, organization and user‐friendliness of a variety of databases designed for the community by investigators essentially unknown to the end users. Work on data structures is therefore vital for biological and medical research but rarely brought into the limelight. Here, for the sake of brevity, we restrict our discussion to prokaryotic genome data.

A further step involving data structuration appears when a subset of genome‐derived data is organized into specialized databases. A first key step in this effort was the attempt to create data structure‐aware databases of proteins, as developed by SwissProt (Bairoch, 1982; Bairoch and Boeckmann, 1991) and the Protein Identification Resource [PIR (Barker *et al*., 1983; Sidman *et al*., 1988)], now united as the UniProt protein annotated database (UniProt Consortium, 2018). Subsequently, query languages devoted to biological sequence management such as the ACNUC language (Gouy *et al*., 1984) were developed. First microbial databases with a minimal data structure were then meant to make whole genome sequences and annotation available to the community (Higgins and Danchin, 1990; Kunisawa *et al*., 1990). A further refinement was based on statistical analyses of the genome structure, leading to the discovery of the key role of horizontal gene transfer in bacterial genomes (Medigue *et al*., 1991). Links with the previously known restriction maps (Kroger *et al*., 1990; Medigue *et al*., 1990) allowed the building up of a more evolved data structure that resulted in the reference Colibri database for the *Escherichia coli* genome (Medigue *et al*., 1993) based on the concept of genomic object as its core item. This database also combined gene annotation with specific methods meant to extract biologically relevant sequence‐based information, as first illustrated in the case of *Bacillus subtilis* (Moszer *et al*., 1999). With the explosion of microbial genome data, it now seems essential that new work begins to build up on these past works [for a discussion see (Borriss *et al*., 2018)] at a time when it appears that interest in building up specialized databases relying on high level data structures is vanishing.

## The annotation process

Annotation is the action of associating specific metadata to entries in a data collection. It heavily depends on the data structure (you do not annotate an object that has not been previously explicitly identified and properly defined). A previous review in this journal identified a standard flow chart for the annotation process (Siezen and van Hijum, 2010). In this section, we restrict our brief review to the automatic annotation of genomic objects (genes and other specific features identified as nucleotide sequences) and some of its consequences. Annotation can be made automatic by chaining a series of methods into a ‘pipeline’ that begins from identifying the genomic object. The most common object is the protein coding sequence (CDS), located within an open reading frame (ORF), *i.e*. with a proposed translation start site. A popular software, prodigal, created at the Department of Energy Joint Genome Institute, is often used to identify bacterial translation start sites (Hyatt *et al*., 2010). However, despite its qualities, it remains imperfect as our knowledge advances. For instance, the genomic G+C content is highly variable, and this may influence ribosome initiation addressing so that rule‐based approaches mimicking biological behaviour should still be explored (Makita *et al*., 2007). Automatic validation of starts can be obtained via alignment of the CDSs of multiple orthologous genes (the initiation codon and ribosome binding sites should be somewhat conserved). Routinely, however, much of automatic annotation still conflates CDSs and ORFs and this is perpetuated by the unfortunate habit of many investigators to use the name ORF instead of CDS. This introduces an ambiguity that frequently contaminates annotation at a very early step, with disastrous consequences downstream, including readily avoidable yet catastrophic mistakes that can propagate to review‐level articles (Kyrpides and Ouzounis, 1998). Automatic identification of translation starts sometimes requires manual annotation. An example of this situation, fairly conserved in Bacteria, is the synthesis of the two subunits of aspartokinase LysC. The alpha subunit CDS (*lysCA*) starts with an upstream AUG codon, while the beta subunit CDS (*lysCB*) starts from a codon located way downstream within the same ORF (Kalinowski *et al*., 1991). In some situations, a programmed frameshift within a CDS will produce two proteins, beginning with the same start site but with a different ORF starting at a specific position. This is the case of the coding region of subunits gamma (DnaX) and tau (DnaZ) of *E. coli* DNA polymerase clamp loader. The tau chain is the full‐length protein; the shorter gamma protein is created from within the tau reading frame by a programmed ribosomal ‐1 frameshift over codons 428 and 429 followed by a stop codon in the new frame (Tsuchihashi and Kornberg, 1990).

Subsequently, the gene sequence is linked to a variety of features, usually meant to propose a function for the gene and related to specific phenotypes (Xiao *et al*., 2015). Most standard annotation efforts rest on pipelines that apply some form of majority rule, where a multiple alignment is performed to derive similar sequences and the most common annotation is accepted (Ekblom and Wolf, 2014). In such a case, there is some room for the person coordinating the annotation process to influence the outcome, in particular via the use of the workflow in a recursive way – that is a first output is used as an input to run the workflow again. This will allow, for example, correction of CDS start sites and changes in the domain organization of the corresponding gene products. This work results in a better annotation, but, of course, this is considerably more time consuming than running an automatic pipeline. An example for bacteria is the workflow used as an input to the MicroScope annotation platform (Vallenet *et al*., 2017).

All this progress in the early sequence annotation methods was achieved using the transitive induction reasoning mentioned above: what looks similar in structure (sequence) should also code for similar functions. This assumption has had a strong implication in terms of improving our biological knowledge. Progress happened mainly when experimental evidence associated a new function to a novel sequence, against a background of a highly variable number of literature reports linked to the first species that were sequenced (Janssen *et al*., 2005). In this context, at a meeting organized in 1991 by the European Union in Elounda (Crete) a completely unexpected observation rocked common knowledge: in the novel sequences present both in a large contiguous piece of the *B. subtilis* genome and in a full chromosome of yeast, more than half of the genes did not look like anything previously known. These Elusive, Esoteric, Conspicuous (EEC) genes suddenly showed that both in terms of sequence and function a vast domain of the gene complement of organisms was entirely unknown (Danchin, 2003). This required specific approaches to fill in the corresponding holes in our knowledge. Despite some drop in the discovery of novel gene sequences, extant metagenomic studies show that the situation did not change drastically since then. This precludes annotation by similarity and we still must annotate newly discovered genes from scratch (Iliopoulos *et al*., 2001). As a consequence, experimentally based evidence remains a critical issue (Chang *et al*., 2016).

In parallel, many experiments revealed that annotation by similarity kept producing errors that percolated throughout databases (Promponas *et al*., 2015). Many of these erroneous annotations still lurk in public databases and have increased in size purely by similarity searches and ‘novel’ assignments (Gilks *et al*., 2005). The combination of ignorance and percolation of errors can be vividly illustrated by the fact that, quite recently, the first synthetic genome of a bacterium (Hutchison *et al*., 2016) was imperfectly annotated despite the importance of the experiment and the quality of the sequencing team. Annotation based on accurately annotated model genome databases improved the outcome, demonstrating that there is a need for such knowledge (Danchin and Fang, 2016). A key question now arises: erroneous annotation is akin to misinformation, systematically leading investigators to explore wrong tracks. To be sure, automatic annotation, which derives from a variety of software and workflows, looks more and more as ‘the wisdom of the crowd’. Yet, we all know (or should be aware of the fact) that knowledge cannot result from an (anonymous) majority vote (McKee and Stuckler, 2017; Lazer *et al*., 2018). Certainly, using a piece of information that is frequently right but often wrong has worse consequences than entirely wrong information (which can be discarded right away).

## Accurate annotation: against majority

Data collection and analysis of genomic sequences, producing automatic annotation based on a majority rule, may result in many pitfalls. An interesting study in the domain of knowledge acquisition and propagation shows how the majority opinion being often wrong (this can be an obvious problem for direct vote in a democracy, as we frequently witness these days) it may still be possible to correct the vote of the crowd in a way that will restore some credibility to the knowledge output (Prelec *et al*., 2017). A widely spread solution (often used for peer review, as we might notice) is to ask all the persons who are involved in producing pieces of knowledge to express their opinion as to whether they are confident in their own response to the task. Unfortunately, this does not work much better than believing the crowd. People tend to be confident in what they say, even when they are wrong. How could we proceed, then? Prelec and co‐workers suggest to ask people to predict among several possible answers what they think will be the majority answer proposed by others and subsequently select the answer that gained more support than expected. Apparently, this ‘surprisingly popular’ approach gives results that are much better than those collected from a direct poll (Prelec *et al*., 2017).

Yet, we are still far from real wisdom and it may be somewhat difficult to implement Prelec's approach in an automatic annotation pipeline. Furthermore, when exploring a new dataset we need baselines to test, using well‐established knowledge and in realistic conditions, whether the outcome of any procedure gives the relevant answer or fails to do so (Danchin and Braham, 2017). Unfortunately, this type of internal control is seldom performed, except perhaps in a variety of learning techniques, where part of the data sample is kept aside and used to test, after the analysis, whether the answer has remained stable. This makes the basis of bootstrapping approaches (Henderson, 2005; Bujkiewicz *et al*., 2013) as well as jackknife validation tests in learning approaches (Chou and Zhang, 1995) or cross‐validation (Arlot and Celisse, 2010). A common pitfall is, again, the fallacy of the average (Denny, 2017): annotating a genomic object using an average set of genome clades is often misleading. Non‐average annotation is particularly sensitive to the large component of genomes that arises from horizontal gene transfer. There is also a considerable sampling bias in the genomes retained as interesting because of our anthropocentric view of what life is (just observe the number of genomes from pathogenic bacteria in a real world where such organisms are in fact a tiny minority).

**Figure 2:**
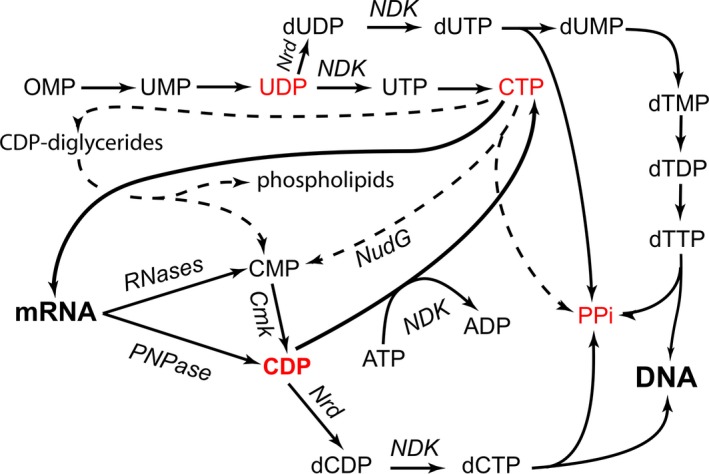
Pyrimidine metabolism and synthesis of DNA precursors. Synthesis of DNA uses NDPs as precursors. Pyrimidine biosynthesis does not proceed *de novo* via CDP, producing directly CTP instead. ATP large excess over ADP does not allow nucleoside diphosphokinase to make CDP so that CDP can only come from RNA degradation, via RNAses and polynucleotide phosphorylase. The maintenance pyrophosphate hydrolase (NudG) would produce CMP from CTP, but it is a moonlighting activity derived from its major substrates which are CTP derivatives modified at position 5 of cytosine.

All these drawbacks have important consequences for the popular domain named ‘systems biology’, when it solely rests on unvalidated gene annotations. Oftentimes, systemic approaches are offered (implicitly) as a way to explore the wisdom of the crowd, in a situation where they stem from very incomplete knowledge. For this very reason, we ought to propose explicit tests for the validity of approaches of this kind, again via the creation of baselines built on knowledge that is certain, but still unfamiliar to the majority of investigators. This requirement should apply at least for system biology studies of metabolism. Here is a straightforward example. DNA synthesis is a growth requirement for all cells. Remarkably, synthesis of deoxyribonucleotides does not follow a path that crowd wisdom would have predicted. Indeed, the synthesis of deoxyribonucleotides starts with ribonucleoside *di*phosphates (NDPs), not *tri*phosphates (NTPs). This represents a parallel thought process with the ‘widely unexpected’ capital of Pennsylvania (Harrisburg, rather than the expected Philadelphia or perhaps Pittsburgh) used in Prelec's study discussed previously. The surprising involvement of NDPs in DNA synthesis turns the corresponding pathways into a simple testbed that allows us to investigate whether a model can predict anything (a common feature of highly redundant models where there are so many adjustable parameters that almost anything can be ‘predicted’). Indeed, using NDPs as precursors has a remarkable consequence for the synthesis of deoxyribocytidine diphosphate, then triphosphate, obviously required to make DNA. It makes DNA synthesis impossible with straightforward metabolic pathways. This is because the *de novo* synthetic pathway of cytosine nucleotides makes CTP directly via ATP‐dependent transamidation of UTP, never going through CDP (Fig. 2).

Where does the necessary CDP come from, then? Any valid model of metabolism should predict that, unless the model includes specific CDP or CMP sources, DNA synthesis should not be possible: nucleoside diphosphokinase, while reversible, is driven in the NTP direction by the large excess of ATP over ADP in the cell (unless perhaps compartmentalized in an ATP‐deficient region of the cell), and the cell maintenance diphosphatase NudG [YnjG (Fujikawa and Kasai, 2002)] which might produce CMP from CTP has been selected for preferring modified nucleoside triphosphates as substrates (Fig. 2). The main solution found by cells is that RNA, a family of macromolecules, not a small metabolite, is involved in the process: DNA synthesis requires RNA turnover. This key fact is rarely taken into account in standard metabolism pathways. Yet, if included in the model, it will provide a straightforward flux diverted to DNA synthesis, via hydrolysis or phosphorolysis (Danchin, 1997). Phospholipid turnover may also contribute to this pathway, but in a limited way. Again, this is seldom a feature identified by metabolic models, which, therefore, show in a vivid way that they are not constructed so as to be falsifiable in Karl Popper's terminology (Popper, 1959 [trad 1935]).

The example above illustrates that specific collection of sequences need to be accurately annotated, with an emphasis on salient, discriminating features. This was the raison d’être of the SwissProt protein database, where protein sequences were directly matched with experimental data (Bairoch *et al*., 2004). Emphasis on connection with properly annotated databases of experimental observations has repeatedly been highlighted but with not always with positive outcomes. Along this line, Roberts and co‐workers have initiated an effort to construct a database of experimentally validated gene annotations, COMBREX (Chang *et al*., 2016). Others have proposed species‐specific, not protein‐centric (re‐)annotation efforts (Ouzounis and Karp, 2002). While this type of validation is key to allow significant progress from data accumulation, only very few investigators have been willing to devote a significant part of their work to this essential activity. A new business model in this field is required to create relevant incentives, so that we reach a sustainable level of experimentally validated annotations.

## Statistical approaches

Even without investigating the way samples are obtained, where an unlimited number of systematic errors are the rule – for example, via biases in collecting sequence data (Mavromatis *et al*., 2012), a typical output of genome studies, big data sampling, does not by itself allow investigators to make discoveries. A vivid illustration of why this might not work is to understand why a neuroscientist attempting to understand the function of a microprocessor, while allowing any kind of big data measurements on its behaviour, would be doomed to fail (Jonas and Kording, 2017). Genome annotation is meant to provide enough information so that, by combining knowledge associated with genes, we could understand how a genome works in enabling the organism (here, essentially the microbial cell) to behave (*i.e*. explore the environment and proliferate) in a variety of niches. Important features are, therefore, the accurate definition of primary data elements (sequences and structures), the identification of consistent clusters of genomic objects (via co‐evolution), as well as connectivity between them (functional complexes).

### Descriptive (exploratory) statistics

As discussed above, we see that the majority rule cannot, by itself, produce reliable annotations for arbitrary collections of sequences. A large number of statistical approaches have been used to improve the quality of the output in parallel with automation. An immediate follow up of the majority rule is provided by Bayesian approaches (Bujkiewicz *et al*., 2013). What these statistical techniques do is to assert, all things being kept equal, that we should find this or that feature with a probability which is subsequently chosen to be compared to a threshold value, commonly accepted by most investigators. While this strategy can be fruitful in many cases, the condition ‘all things being kept equal’ implies that this approach cannot lead to discovery, revealing unexpected views on the role of genomic objects, in particular their connection to other such objects. It is also unfortunate and commonly observed in biology that things are rarely kept equal, except in highly related organisms and environments. In a way, living cells are extremely ‘imaginative’, challenging our common sense expectations. Just observing the shape of bacteria (expected to be quite ‘uninteresting’), we marvel at their bewildering variety of forms (Kysela *et al*., 2016). Yet, this seems to be due to a specific constraint: spherical membranes tend to be growing with a growth rate based on the square power of the cell's radius, while the metabolic pressure creating a cytoplasm goes as the cube power of this radius (for a sphere). As a consequence, membranes tend to develop into shapes that are far from that of a sphere (Harris and Theriot, 2016). Subsequently, shapes, and odd shapes in particular, will open the door for novel functions, for example*,* see *Thiovulum majus* which uses an unusual hydrodynamic power to get the environmental water medium to approach the cell and feed it (Petroff and Libchaber, 2014).

**Figure 3:**
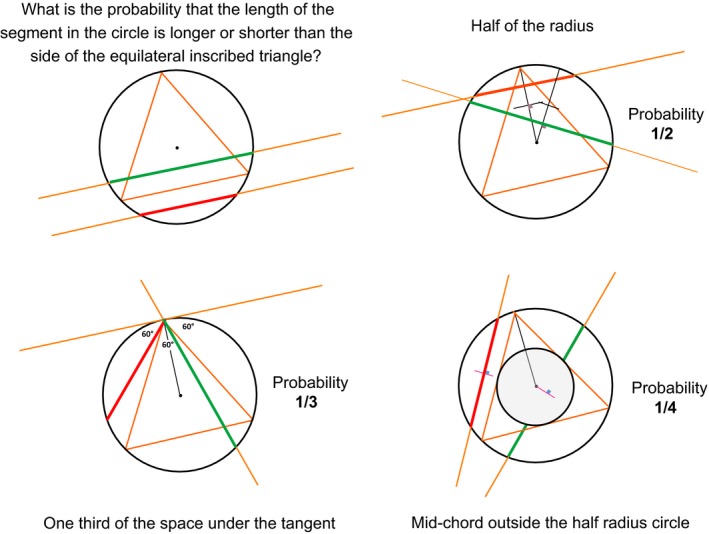
Bertrand's paradox. Finding a solution to a straightforward probability question may depend on the context. Investigating the probability for a line to cross a circle with a length smaller than that of the side of the inscribed equilateral triangle gives different answers if one considers intersection with a radius of the circle (1/2), position with respect to a summit of the triangle (1/3) or intersection with the homothetic circle with half the circle's radius (1/4).

Bias in data choice (our anthropocentric view of microbes highlights pathogens) as well as bias in data importance (abundance of species*,* versus relevance of species) plagues all statistical approaches. There are also many biases in statistics because a probability depends on the model of ‘randomness’ which is used in the background, often unknown to the authors (see Bertrand's paradox, Fig. 3). Another difficulty, rarely realized by many, is that the vast majority of statistical approaches rely on specific properties of the data sets. As a matter of fact, data samples are expected either to differ because of a large variety of additive causes, or of multiplicative causes. The former case should give a normal (*i.e*. Laplace‐Gauss) distribution of data items in the dataset, while the latter should give a log‐normal distribution. As a consequence, one would expect that investigators begin, before embarking on any type of statistical analysis, by plotting their data distribution and checking whether they are normal or log‐normal, completely haphazard or belong to one of the many other distribution shapes that have been explored by statisticians. This simple but essential step is rarely documented in articles reporting on big data collection. Furthermore, data pre‐processing is another step of critical importance (Karaman, 2017). While rarely made explicit, it should be an absolute requirement for all big data collections.

**Figure 4:**
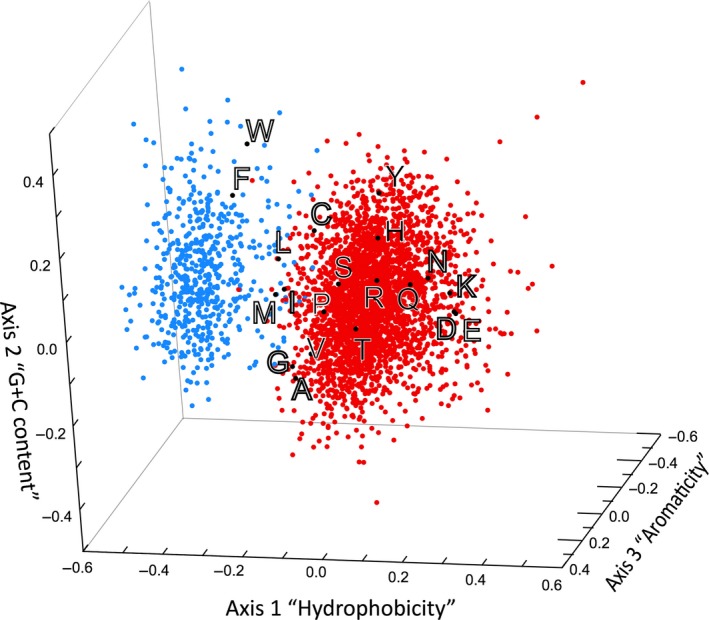
Correspondence analysis (CA) of the amino acid composition of a proteome [redrawn and modified from (Riley *et al*., 2008)]. In CA, the data and their qualitative variables can be swapped so that they can be visualized within the same spatial representation. Here, each dot represents a protein extracted from the proteome of a bacterial species, and spatial distribution of the amino acids is superimposed on the corresponding cloud of points that differentiates between membrane proteins (blue) and the rest of the proteome. It can easily be observed that large hydrophobic amino acids (phenylalanine, tryptophan, methionine and leucine) are strong markers of membranes, while charged and hydrophilic residues (aspartate, glutamate, lysine and asparagine) would be concentrated within cytoplasmic proteins.

To make the most out of data its connection to metadata is a necessity. This is often displayed as involving matrices made of *p* items endowed of *q* qualities (commonly named variables), with *p* usually very large (or in ‘omics’ data often with *q* >> *p* as well). A good many multivariate analysis approaches exist to explore this type of data (Lebart *et al*., 1984; Neely *et al*., 2012, 2013). Their role is to provide a statistical procedure to explore the data in a pertinent way. In exploratory statistics, the aim of the method is to try and reduce the number of qualities (viz. variables) that contribute to the data order. Mathematical methods used to this end rest on specific hypotheses about the distribution of entities within the data. Normal or log‐normal datasets have been used to generate a variety of multivariate analyses based on this widespread yet limited statistical constraint. Among those, Principal Component Analysis (PCA) is quite popular. With this technique, the measure that monitors the distance between entities of interest submitted to analysis commonly uses the variance of each quality or of a linear combination of qualities as a normalization factor. This is a convenient measure, but with only indirect links to the investigated processes (Katagiri and Glazebrook, 2009). To be sure, this does not always fit with the actual information embedded in the data (Benzecri, 1973). In contrast, Correspondence Analysis (CA) makes use of *chi‐square* distances for classification of objects without *a priori* knowledge of the classes (Hill, 1974). This allows introduction of a valid information measure between characters (Danchin, 1996), which lacks in PCA (Fellenberg *et al*., 2001). CA should thus be the preferred method for the study of large data sets that comprise qualitative variables linked to quantitative data. The measure used, indeed, creates a dual space that allows investigators to consider items and qualities as equivalent, so that the same output can be used to visualize simultaneously individual items and their qualities (Fig. 4). This is of considerable help to associate biological knowledge to large datasets.

Datasets often deviate from the normal or log‐normal distribution. In particular the distribution may be asymmetrical, have long tails or both. Asymmetrical heavy‐tailed distributions may follow Cauchy's law that accounts of the ratio of two independent variables, and this is equivalent to Student's variable of degree 1, comparing well with standard statistical tests (Zimmerman and Zumbo, 1990). Gumbel's law is used for analysis of the distribution of extreme values in a data sample. It has been shown to be important to understand the validity of sequence alignments compared using the Smith–Waterman algorithm (Comet *et al*., 1999; Bacro and Comet, 2001). In metagenomic studies, the zero‐inflated Poisson model accounts for random events containing excess zero‐count data (Liu *et al*., 2016). Weibull's law is used for samples monitoring entities that degrade with time, become better with time or exhibit only a random degradation pattern. It is used for data describing adhesion and transmission of bacterial communities, as well as reliability of items qualities in the course of time (Gavrilov and Gavrilova, 2001; Heathcote *et al*., 2004; Gusnaniar *et al*., 2018). Typically, one should decide of a specific statistical multivariate analysis on a case‐by‐case basis, remembering that the outcome of a study will heavily depend on this critical choice.

Yet, other methods do not require normality. Let us point out just one of those here, as it may help us make some sense from biological data even in the absence of well‐formulated hypotheses. Independent Component Analysis (ICA) is a dimension reduction technique (Jutten and Herault, 1988), sometimes nicknamed ‘the cocktail party’ multivariate analysis. The idea is the following. Consider a cocktail party with some 1000 guests and five microphones: would it be possible to reconstruct the conversations of some of the guests? The answer is positive. It is based on the fact that conversations are essentially independent from one another (except for loudness, which is obviously correlated to the number of guests but does not affect the independence of the conversations otherwise) so that one can use the existence of independent factors in multivariate data and decompose an input data set into statistically independent components. Interestingly, this fits with data that are not distributed along normal or log‐normal patterns. ICA can reduce the effects of noise or artefacts of the signal and is ideal for separating mixed signals. Widely used for image analysis, ICA was used for microarray analysis to extract expression modes of genes (Liebermeister, 2002; Zhang *et al*., 2005). A strong reason for including it here is that the validity of the method in molecular biology could be confirmed via its substantiation in bacterial transcriptome data. Indeed, genes belonging to operons are found with ICA to cluster together despite the fact that the operon information has not been used as input or a feature of the data, providing thus an independent internal check of relevance (Carpentier *et al*., 2004).

These methods permit investigators, under some conditions, to build up valid hierarchical clusters grouping together items with similar properties (linked to the variables in the study). The underlying idea, due to Vicq d'Azyr (Vicq d'Azyr, 1792), is that one can construct pertinent classes where objects do not share simultaneously all the characters defining the objects in a given class and nevertheless belong to the same class because they share most of the characters defining a class (Benzecri, 1973). Many approaches can be used to build up clusters. A method used sometimes, such as conjunctive consolidation, arranges data into clusters in a semi‐automated way (introducing knowledge of the investigator). It has been named ‘multivariable analysis’ because it uses data from multiple variables to ‘explain’ the behaviour of a very small number of outcomes (Neely *et al*., 2013). In general, however, investigators aim at having an automatic way to construct clusters of relevant biological importance. Perhaps the most often used techniques are those related to K‐means, where, starting from a random set of possible centres of gravity of clusters one progressively moves them to allow the formation of consistent clusters. The investigator can influence the outcome by deciding, at the outset of the computation, the number of expected clusters (Do and Choi, 2008). Performing this computation while increasing this number allows one to find a situation that can be regarded as optimal, considering the dataset and based on measures such as the silhouette, a measure of clusters cohesion (Kim and Kim, 2017). Labelling the data within the clusters following their construction with metadata that have not been involved in the computation allows one to examine whether they are biologically relevant, paving the way to inferential statistics semi‐supervised *K*‐means (Bair, 2013).

### Inferential statistics

Once the data are structured, it may be of interest to go further. The goal of a multiple logistic regression is to find an equation that best predicts the probability of a value of the *y* variable as a function of the *x* variables. A standard inference procedure measures the independent variables on a new individual and estimates the probability of it having a particular value of the dependent variable. This can be evolved further, using multiple logistic regression to understand how the independent variables are functionally linked to the dependent variables, in an attempt to discover what might cause the probability of the dependent variables to change. However, one needs to be very careful because inferences are plagued with numerous pitfalls (Shen *et al*., 2014). Finally, big data studies may sometimes end up in a situation exactly opposite to that explored by usual multivariate analyses: there may appear a multiplicity of features, much larger than the items in the sampling data set; this yields overfitting with multiregression techniques. Partial Least Square (PLS) regression is an approach that avoids the overfitting outcomes of most other approaches in this situation (Fort and Lambert‐Lacroix, 2005; Worley and Powers, 2013).

At this point, most statistical approaches have been used to provide a diagnostic structure of the data. This diagnostic can be extended to the construction of models that will illustrate in a predictive way the behaviour of the data if more data are included. However, this will not provide an explanation of the reasons why the data have been clustered in this or that form. Further knowledge must be included to advance a preliminary outcome, in particular via supervised learning. As an example, random forests build up many decision trees and end up in the majority rule (remember the caveat previously discussed), choosing the tree that got the most votes (Breiman, 2001). The process produces interpretable models that are invariant under scaling, robust to inclusion of irrelevant features but can overfit (low bias and very high variance). Random forests, at the expense of interpretability, also support decreasing the variance by aggregating votes of many trees each built on random samples with replacement of the training set. Support Vector Machines (SVM) are yet another supervised learning algorithm that finds a hyperplane that represents the largest separation (also called margin) between the samples of two classes (Ben‐Hur *et al*., 2008). The SVM technique relies on the definition of distances (often Euclidian) to output a model building an hyperplane that has the largest distance to the nearest training data point of any class. Such max‐margin hyperplane is completely determined by those vectors which lie nearest to it and are called ‘support’ vectors.

## Sequence, structure, function

A prevailing belief across modern molecular biology is that a gene sequence will define the structure of the gene product and that structure, in turn, will designate a unique function. Oblivious to those who subscribe to the above view is that this logical sequence assumes that living organisms have somehow been designed to perform what they do. In other words, the strong version of the motto ‘from sequence to structure to function’ directly leads to intelligent design. By stark contrast, we stress here that annotating properly gene sequences must start with a first constructive principle of no intelligent design in biology. To some extent unexpectedly, this precondition has many surprisingly positive consequences in terms of allowing us to understand biological functions. If one accepts this as a principle (‘there is no design to be expected’, somewhat in the way of the common principle: ‘the laws of physics are the same everywhere in the universe’, there is no centre, there are no special laws), we are led to a remarkable set of practical consequences. Some illustrations follow.

## Fixing carbon dioxide

Biology obeys the laws of physics and, in particular, the second principle of thermodynamics, which states that every material system will tend to explore all reachable space positions and energy levels – this exploration has often been misidentified as implying disorder, forgetting that order requires an observer to distinguish it from the background (Danchin, 2003). In addition, living organisms manage information as an authentic currency of physics (Landauer, 1996). Organisms use information to channel the exploration of the universe within the borders of defined material systems. To define this trajectory, they use Landauer's principle that establishes that information can be created without energy consumption (Landauer, 1961). To follow suit and carry on, living organisms must, however, use energy to erase the memory of the processes they used to create that particular information – that is reset it to its original state. This implies that creating functions can be a straightforward, yet highly unforeseeable process. This view should be developed further, as ‘function’ is a very deep concept (Allen *et al*., 1998). Here we use the concept is the fairly loose way commonly used by biologists.

Consider as a first example the fact that living organisms are based on carbon chemistry, together with the information that carbon dioxide is a readily available, unused, carbon store. ‘Fix carbon dioxide’ becomes an obvious function of immense interest for life to develop. Yet, this does not tell us how this function could be implemented. Any function is built up from material systems, so that biological systems are compelled to use what material entities they have at hand or in fact need to discover. This straightforward observation makes one realize that the obligatory interaction of this information management with matter leads necessarily to a broad range of opportunities. A stark analogy is a stranded person on a desert island, a result of a shipwreck disaster: all available means, materials and options are considered to achieve survival, no matter what the design limitations may be. This is the ‘function first’ driving force for survival and in fact reflects much of the tinkering aspect of biology. Even a general informational principle (such as the rules and processes involved in coding nucleic acids into proteins, the Central Dogma of Molecular Biology), while effective after the fact, is constrained to be embodied into highly specific material entities – in our case the quasi‐universal rules of the genetic code. And since no one has ever designed this process, this embodiment is, within the constraints of matter, of course, purely ad hoc. It makes use of all means at hand.

Another striking illustration is Ribulose Bisphosphate Carboxylase Oxygenase (RuBisCO), the most abundant enzyme on the planet, responsible for the fixation of carbon dioxide (Feller *et al*., 2008). This enzyme, recruited long before photosynthesis (Yokota, 2017), is still today very slow despite its functional necessity and at least a billion years of evolution. It is plagued by a parasitic side reaction that uses oxygen, within the very organisms that produce it while fixing carbon (Erb and Zarzycki, 2018). In other scenarios of structure recruitment for carbon fixation, more efficient but still slow systems have naturally evolved at least on five further occasions, recruiting widely different enzymes (Fuchs, 2011). Their overall lack of efficiency motivated a sizeable effort of synthetic biology, which took up the problem from scratch, but using the human brain to make it via, this time, an intelligent design: A seventh, synthetic, fixation process has recently been developed *in vitro* (Schwander *et al*., 2016). In summary, the basic requirement of important functions results in convergent evolution via the recruitment of pre‐existing structures, when available. With this example, we can understand that such evolution processes have strong bearings on genomic object annotation, where we need to think ‘function first’ rather that ‘follow the crowd’.

## Any important need elicits an adaptive function

As other great apes, human subjects long used to eat with their hands. However, as the role of using garments to dress oneself began more and more common, in parallel with a role in expressing a social status, it quickly became important that clothes needed to be clean and shiny. To avoid soiling our hands we used specific tools: a large leaf, a pair of chopsticks or spoon and fork, even combining them together. A similar protection against dirt is prominent in the construction of bicycles or cars. In the same way, cells tend to create new entities in order to improve basic functioning (Acevedo‐Rocha *et al*., 2013).

Let us consider cooking food fast, with a minimum energy cost. Look at a pressure cooker. Its function is clear, as it uses the thermodynamic role of high temperature to accelerate considerably the cooking of ingredients of biological origin. This has a trade‐off. Maintaining a high temperature in a confined vessel will result in high pressure. Usual cookers would not withstand pressure, so that one needs to build strong enclosures. But this results in creating a bomb‐like contraption, in any event of fast or uncontrolled temperature rise. Hence it is necessary to build both a regulation system (this will be a regulatory valve) and an emergency safety valve, that will release some steam above a certain pressure threshold. We observe the same principle in cells, which multiply in a variety of environments, possessing the exact counterparts of these valves (Danchin, 2009). Sugar transport is usually very efficient and might increase the internal pressure to a deleterious level. Also, a variety of metabolites and ions may suddenly be absent in the environment, placing the cell in low osmolarity: this would result in membrane disruption, unless specific safety valves, the mechanosensitive channels where opening pores in the membrane is reminiscent of the iris diaphragm movement (Zhang *et al*., 2016).

Another common example of a key function conserved in highly reduced genomes is that of the final steps of RNA degradation. While a variety of endo‐ and exo‐nucleases degrade macromolecules of RNA, binding of the substrates decreases as their length shortens all the way down to approximately 5 nucleotide‐long nanoRNAs. These molecules can be potentially highly toxic compounds as their size fits the transcription and replication ‘bubble’. Hence, there is a need for a nanoRNase activity, which, indeed is present in all cells. However, the structure of these enzymes does not arise from a common descent (Liu *et al*., 2012), substantiating again the role of convergent evolution based on function‐based recruitment of material activities. This same observation, that any need creates a function, is made everywhere, again and again, and this makes biology boundaries so difficult to conceive. On the one hand indeed, biology is built up from deep concepts (laws), remarkable for their abstraction – for example rewriting of a text and recursion, systematic development of algorithms. Simultaneously, on the other hand, each living organism has to develop within the material world as a concrete realization of those deep and abstract laws.

## The future: expert systems for genome annotation

Expert systems are meant to mimic automatically the reasoning of an expert (Duda and Shortliffe, 1983). Expert genome annotation systems combine rules, incomplete knowledge and contradictory evidence [see, e.g. (Cadag *et al*., 2007)]. Ideally, such systems should associate deduction (applying existing rules), induction (building new rules, e.g. exploring neighbourhoods) and in a most unlikely development, abduction [suggesting explanations based on serendipity, see (Ferneda *et al*., 1995) and references therein]. The ultimate goal, still not available today for a robust implementation of automated annotation, should embody the hypothetico‐deductive approach, that chains a hypothesis (generated by the context and pre‐existing knowledge), deduces predictions from the hypothesis and tests whether the predictions allow for identification of an unexpected object or conflict with these predictions in order to disprove some of its features, leading investigators to amend or even discard the hypothesis. As stressed previously, to make the most of existing knowledge, these methods should be based on functional analysis (*i.e*. start from a summary of what could be a living organism propagating in a particular environment), in a way reminiscent of the SynBio engineering reasoning, to identify contradictions and propose experiments (*in vivo*,* in vitro*,* in silico*) to the annotator.

### The hypothetico‐deductive approach illustrated

In a model paper describing workflows to improve annotation of paralogues, de Crécy‐Lagard and co‐workers summarized the best of what can be done at present using standard approaches [available software web services in particular (Zallot *et al*., 2016)]. This emphasis on information incorporates constraints such as the impossibility to make DNA without turnover of RNA, discussed previously. It also rests on the logic of metabolism: based on enzymes paralogous to those present in pre‐existing pathways, development of novel pathways involving related compounds is pre‐set for emergence of fully functional pathways [paralogous metabolism (Chan *et al*., 2014)]. An example of this situation is the *yxeKsnaByxeMNOsndByxeQ* operon of *B. subtilis*, which metabolizes sulfur‐containing metabolites generated by accident (Niehaus *et al*., 2018) and involve a protection (by acetylation, SnaB)/deprotection (by deacetylation, SndB) step. Another example would be the outcome of protein sequence multialigments based on the presence of indels rather than amino acid similarities (Khadka *et al*., 2017). Global discrepancies then suggest reassignment of protein function, which can be subsequently tested experimentally [e.g. illustrated in the differentiation of agmatinases and arginases (Sekowska *et al*., 2000)]. Yet another approach would be to look for ‘missing’ genes coding for essential functions. This idea was used in the identification of several unknown functions coded by a synthetic genome (Danchin and Fang, 2016). In the same way, the fact that the lysine biosynthetic pathway missed a key enzyme in *B. subtilis*, together with the observation that protection against misuse of non‐proteinogenic amino acids is performed by acetylation in *B. subtilis* rather than succinylation as in *E. coli* (Bastard *et al*., 2017), led us to make the hypothesis that protein PatA, annotated as an aspartate aminotransferase (Berger *et al*., 2003), might be the missing *N*‐acetyl‐l,l‐diaminopimelate aminotransferase DapX that we identified correctly by subsequent experiments (Borriss *et al*., 2018).

### Induction: analysis of neighbourhoods

Induction can proceed via the analysis of co‐evolution. An obvious way to take evolution into account is to look for conserved syntenies and combine them with metabolic knowledge for example. The approach has been used in the CanOE strategy (Smith *et al*., 2012), where it allowed investigators to infer the anaerobic allantoin degradation pathway in *E. coli* K12. A further improvement is to introduce phylogenetic distance: a feature that has been conserved in highly divergent organisms should have more weight than when present in organisms that are close to one another (Engelen *et al*., 2012). This inductive reasoning is fairly rewarding for predicting functions, but it is obviously very sensitive to horizontal gene transfer (HGT). As a consequence, genomes should be, prior to exploration, split into consistent subgenomes that would take HGT into account (Doolittle and Brunet, 2016).

In short, a living organism cannot be summarized as the collection of all its genes and gene products, as we need to know their structural and functional relationships as well. Knowledge of entire genome sequences is a unique opportunity to study the relationships between genes and gene products at the level of the cell, the unit of heredity. In most cases, we ignore what relationships are involved; however, we know that they do exist and have only a partial view of them via high‐throughput experiments. Remarkably, this very knowledge provides us with a methodological handle to study them. Using ‘neighbourhood’ in the broadest sense provides us with a way to make fruitful inferences via similarity searches (Benson *et al*., 1996; Nitschke *et al*., 1998). When big data are connected to neighbour information, we may proceed by induction and extract relevant information about their biological meaning. Neighbours should be considered in the broadest sense, making reference to all the items, of all possible kinds (objects as well as processes), that can be related to a particular item. As in all types of data exploration it is important, first, to organize the data along a particular pattern, providing the study with a data structure (Bacon and Anderson, 1986; Clift *et al*., 1986; Lawrence, 1986). The first and most intuitive relationship between two genes is their proximity in the chromosome. Here, neighbours are genes that preserve synteny (Bentley and Parkhill, 2004). Although the concepts of operon, or in a broader sense, of pathogenicity islands, are clearly related to such proximity, this kind of relationship is far from sufficient to explain functional relationships between genes. Furthermore, while genes come together during horizontal gene transfer, there is a systematic disruption that goes on as organisms evolve via insertion/deletion of genes, possibly favouring coexpression of functionally related genes (Fang *et al*., 2008). In addition, gene loss patterns – analogous to the study of indels in multialignments, reveal relationships that cannot be captured by presence alone (Kunin and Ouzounis, 2003).

As another example of an important neighbourhood, phylogenetic proximity reveals proximity due to evolution from a common ancestor. While highly significant, it is very important to find independent ways to separate between orthologues (with conserved functions) and paralogues, with different, sometimes widely divergent, functions (Brown and Babbitt, 2014). Other neighbourhoods may involve metabolism of nucleotides, patterns of nucleotide composition (Dufraigne *et al*., 2005)], or amino acids (Pascal *et al*., 2006). Specific neighbourhoods also relate genes contributing to common metabolic pathways. Furthermore, metabolites shared between pathways are also creating specific links between genes. This creates another large family of metabolic neighbourhoods. We must also explore neighbourhoods based on biases in the genetic code usage. Analysis of this type of neighbourhood is highly rewarding in terms of functional inferences [some illustration in (Nitschke *et al*., 1998; Szklarczyk *et al*., 2017)]. Another useful trend, certainly not utilized as it should be, is proximity in the literature. To be sure, various investigators have reasons to put together particular genes in the article they write, and this often might signify some type of deep, not immediately obvious, connections. The idea of proximity in articles was at the origin of a smart feature of the entrez software (Benson *et al*., 1996). It has also been at the origin of much research based on automatic exploration of the literature – here too, not used enough in biological research, other than the identification of plagiarism (Nawab *et al*., 2014). The idea that two genes can be linked because they are cited in the same bibliographical source lies at the heart of the iHOP software resource (Hoffmann and Valencia, 2005).

### Abduction: extracting information from phenotypes

Serendipity is a common precondition of discovery. It is therefore important to invent approaches that would increase the chances of finding something without a clear path for discovery. The discovery of cyclic‐di‐GMP as a widely present second messenger is a case in point. As a general path, we may look for a function, take the cognate genes, compare them with counterparts, see with which other CDSs they co‐evolve, express and purify the corresponding proteins, find their substrates and regulators. Then, if something unexpected shows up, start around that particular point. This is exactly what happened with cyclic‐di‐GMP, with a long lag between the identification of the molecule and its involvement in general regulatory processes (Romling and Galperin, 2017). This was followed by serendipitous detection of cyclic‐di‐AMP and cyclic‐di‐GAMP (Davies *et al*., 2012; Hallberg *et al*., 2016). In the same way, we accidentally observed that growth of *B. subtilis* on S‐methyl‐cysteine in the presence of dioxygen was abolished when deformylase DefB was inactivated. This led us to understand that, contrary to the expectation that the sulfur atom would be oxidized in the degradation pathway, the methyl‐ group was oxidized, unravelling a completely novel degradation pathway (Chan *et al*., 2014).

We end up with a challenge to the reader, as a way of encouraging serendipitous discovery. Here is an example, reminiscent of the story of cyclic‐di‐GMP before its discovery. The PhoU protein is widely present in bacteria and frequently co‐regulated with the *pst* and *pho* genes involved in regulation and in transport of phosphate. It has neither features of a regulator nor of a transporter subunit. It is widely present in bacteria. Remarkably, it is uniquely absent from *B. subtilis* or *B. pumilus*, but present in *B. cereus*,* Listeria*,* Clostridia* sp. and even Mollicutes with their streamlined genomes. Transcriptome studies did not provide further insight, except to emphasize the importance of the protein. Mycobacteria have two PhoU paralogues. In *Mycobacterium smegmatis,* the absence of the PhoU proteins resulted in a toxic phosphate uptake by the Pst system (Brokaw *et al*., 2017). Interestingly, impairing translation triggers a phosphate starvation response (Pontes and Groisman, 2018). A plausible conjecture is therefore that PhoU is an enzyme, producing yet another metabolite, presumably containing phosphate, that interferes with homeostasis of phosphate metabolism associated with the ATP control of translation, perhaps via translation throttle EttA (Boel *et al*., 2014).

## Conclusion

The future of synthetic biology and biotechnology in general rests on accurate biological knowledge. Genome annotation is a critical step for gene‐based discoveries at the time of big data metagenomics. While a wealth of automatic annotation pipelines are developing, it becomes crucial that their input is not systematically flawed: ‘garbage in, garbage out’. Maintenance of knowledge bases collecting trustworthy information about model organisms – the list of which being enhanced in a judicious way – is key to avoid spending huge amounts of human and financial resources to no avail. Relevant business models need to be invented to attract scientists into contributing to educated gene annotation and construction of reference knowledge bases.

## Conflict of interest

None declared.
